# Association between maternity leave policies and postpartum depression: a systematic review

**DOI:** 10.1007/s00737-023-01350-z

**Published:** 2023-07-17

**Authors:** Liliana Hidalgo-Padilla, Mauricio Toyama, Jessica Hanae Zafra-Tanaka, Alejandra Vives, Francisco Diez-Canseco

**Affiliations:** 1 CRONICAS Center of Excellence in Chronic Diseases, Universidad Peruana Cayetano Heredia, Lima, Peru; 2 Departamento de Salud Pública, y CEDEUS, Pontificia Universidad Católica de Chile, Santiago de Chile, Chile

**Keywords:** Postpartum depression, Maternity leave, Health policy, Employment rights

## Abstract

**Purpose:**

Working mothers are at greater risk for postpartum depression. Maternity leave characteristics, including length, wage replacement and employment protection, could have relevant implications for mothers’ mental health. We propose to explore whether there is an association between maternity leave characteristics and postpartum depression.

**Methods:**

We conducted a systematic review searching for randomized controlled trials, quasi-experimental, cohort or cross-sectional studies on five databases using search terms including maternity and parental leave and depression, as well as references in relevant articles. We identified 500 articles and included 23 of those. We used the EPHPP Quality Assessment Tool for Quantitative Studies to assess the quality of the studies.

**Results:**

Paid and longer maternity leaves tend to be associated with a reduction of postpartum depression symptoms in high-income countries. No studies explored the association between employment protection and postpartum depression. The quality of studies ranged from strong to weak, mostly influenced by study design.

**Conclusion:**

More restrictive maternity leave policies tend to be associated with higher rates of postpartum depression, although more research needs to be conducted in the Global South.

**Supplementary Information:**

The online version contains supplementary material available at 10.1007/s00737-023-01350-z.

## Introduction

Postpartum depression is defined as an episode of major depressive disorder within the first year after childbirth (Munk-Olsen et al. 2006). It is estimated that postpartum depression affects 13–19% of mothers who have given birth (O’Hara and McCabe 2013; Liu et al. 2022). Working mothers, who today represent a more significant part of the workforce than in previous generations (Buzzanell and Liu 2007), are at greater risk of postpartum depression due to sleep deprivation, the demands of caring for an infant, and the inability to engage in health promotion activities because of competing demands from home and work (Selix and Goyal 2015). Since depression is among the costliest health-related conditions for employers (Stewart et al. 2003) and the burden of depression is 50% higher among females (Kessler et al. 2005), it is crucial to identify the relationship between maternity leave policies and postpartum depression.

As stated by the International Labor Organization (ILO), “paid maternity leave is a core element of the health and economic protection of women workers and their children over the perinatal period” (Addati et al. 2014). Maternity leave policies offer formally employed women time off from work before and after childbirth. This period allows them to adapt to motherhood, care for their newborns and helps prevent or reduce the stress of their new responsibilities. Maternity leave characteristics, such as length, wage replacement (paid, partially paid or unpaid leave) and employment protection (guaranteeing the right to return to the same or equivalently paid position at the end of maternity leave) during and after leave, could have relevant implications for mothers’ physical and mental health (Andres et al. 2016; Staehelin et al. 2007; Van Niel et al. 2020).

To our knowledge, three reviews explore how maternity leave affects the mothers’ depression outcomes, among other health outcomes, but none of those specifically focused on how specific maternity leave characteristics are associated with depression. Two of these reviews showed that shorter maternity leaves are associated with a greater risk of postpartum depression (Andres et al. 2016; Staehelin et al. 2007), while the third found that paid maternity leave decreases postpartum depression (Van Niel et al. 2020). However, most of the studies were conducted in the United States of America (US), showing a lack of evidence from other settings. Furthermore, these articles focused only on one of the aspects of maternity leave –either length or wage replacement– and neither assessed the association with employment protection.

Although maternity leave policies exist worldwide, their characteristics vary widely. The ILO recommends maternity leave to be employment-protected, paid and no shorter than 14 weeks, with postnatal leave no shorter than six weeks (International Labor Organization 2000). By 2021, paid maternity leave ranged from 2 weeks to 3 years, with 118 countries guaranteeing at least 14 weeks of paid maternity leave, while 5 did not have any form of paid maternity leave (World Bank 2022).

For example, in the US, the Family and Medical Leave Act (FMLA) of 1993, 29 U.S.C Sect. 102 (a) (1) (A) and (B) is a national policy to support employees due to family and medical reasons. In the case of childbirth, the FMLA provides up to 12 weeks of unpaid, employment-protected leave per year for childbirth or adoption and for caring for an ill family member. To access this benefit, mothers must have worked for an employer with 50 or more employees for a minimum of 1250 h in the previous year (Simmons 2000). Some states have laws with different characteristics. For example, Minnesota provides 12 weeks of unpaid leave but is less rigorous in the requirements (Dagher et al. 2014), and California provides up to 8 weeks with 60–70% wage (Employment Development Department n.d.).

In Peru, where the research team is based, according to Law 30,367, mothers of newborn children have the right to take up to 14 weeks of employment-protected leave, equally divided between antenatal and postnatal leave (El Peruano 2015). Maternity leave includes full wage and a nursing subsidy. To access this benefit, mothers must have contributed to social security for three consecutive or four non-consecutive months, have been formally employed at conception, and maintain their job during their leave. However, women with informal jobs are ineligible, which is concerning since it is estimated that 72% of Peruvian workers have informal jobs (Séruzier et al. 2014), which is also common in other low- and middle-income countries (LMICs) (International Labor Organization 2018).

A better understanding of the impact of maternity leave on postpartum depression could shed light on ways to improve this policy and, as a result, women’s mental health. Therefore, we aim to identify the relationship between the characteristics of maternity leave policies and postpartum depression according to the existing evidence with the proposed research question: Is there an association between maternity leave and its characteristics (length, payment scheme and employment protection) and women’s postpartum depression?

## Materials and methods

### Search strategy

The search was conducted on November 18, 2021, on PubMed, PsycINFO, EMBASE, CENTRAL and Global Index Medicus. The search strategy included terms related to maternity leave, postpartum depression and maternal health and wellbeing. Although the review focuses on maternity leave, we opted to include in the search broader terms (e.g. parental leave, family leave) to ensure all articles referring to women taking maternity leave were included. The search strategy did not restrict by year of publication or language. The full search strategy is available in Supplementary file 1. Relevant studies included in the reviews mentioned above were also considered for selection.

We included quantitative studies (i.e., Randomized Controlled Trials (RCTs), quasi-experimental, cohort and cross-sectional studies) assessing postpartum depression. Qualitative studies, grey literature, editorials, letters, case reports, reviews and conference abstracts were excluded. We also excluded articles that explored only paternity leave or that did not show results specifically for women using parental leave. We included studies published in English or Spanish.

Two independent researchers conducted the study selection in a two-step process (titles and abstracts, and full text) using Rayyan software (Johnson and Phillips 2018). Inconsistencies were discussed with a third researcher to achieve consensus on a final decision.

### Data extraction

The data extraction was conducted by the two independent researchers who also conducted screening using an Excel spreadsheet. For each included study, extracted data included the author, publication year, title, country, study design, source population, inclusion and exclusion criteria, sample characteristics (age, education level, socioeconomic status, relationship status, job type and childbirth/motherhood characteristics), maternity leave characteristics (leave length, wage replacement, and employment protection), policy characteristics (policy reach and requirements to access leave), and effect measures of depression outcomes depending on how articles presented them (e.g. mean difference, confidence interval, standard error).

### Data synthesis

We conducted a narrative synthesis of the effects of each of the three maternity leave characteristics. Initially, we planned to conduct a meta-analysis; however, we found great heterogeneity between the exposures and outcomes reported in the studies. For example, length of leave was measured as either a continuous variable in days or weeks or as a dichotomous variable with cut-off points that varied widely between studies. Additionally, inadequate reporting of the results prevented us from conducting the analysis. For example, some studies presented only effect size, but not standard errors or confidence intervals.

### Study quality assessment

The quality of quantitative studies was assessed using the EPHPP Quality Assessment Tool for Quantitative Studies (Thomas et al. 2004). This tool was developed to assess public health intervention studies on various topics and is an adequate instrument for quality appraisal (Thomas et al. 2004). The protocol was pre-registered on PROSPERO to ensure transparency (ID CRD42021290413).

## Results

**Fig. 1:**
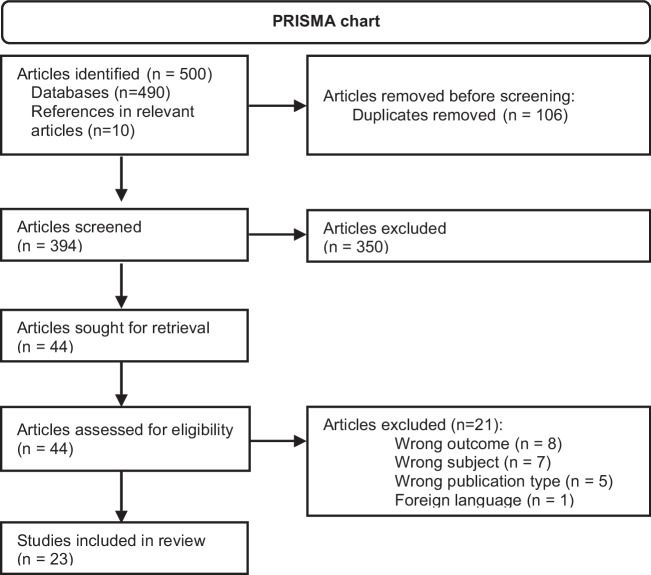
Flocwchart of study selection

The number of articles identified, screened, and included, and the reasons for exclusion, are detailed in Fig. 1 following the recommendations of the Preferred Reporting Items for Systematic Review and Meta-Analysis (PRISMA) Statement (Page et al. 2021). The agreement level between raters was 92% at the title and abstract stage and 100% at the full-text review stage.

Twenty-three articles were included to explore the association between maternity leave characteristics and postpartum depression. Studies were conducted in the US (*n* = 18) (Chatterji and Markowitz 2005, 2008; Clark et al. 1997; Dagher et al. 2014; Dundon et al. 2021; Feldman et al. 2004; Gjerdingen et al. 1991; Hwang et al. 2021; Hyde et al. 1995; Jou et al. 2018; Klein et al. 1998; Kornfeind and Sipsma 2018; Mandal 2018; Petts 2018; Richman et al. 1991; Shumbusho et al. 2020; Stack et al. 2018, 2019), Canada (*n* = 2) (des Rivières-Pigeon et al. 2001; Baker and Milligan 2008), Australia (*n* = 1) (Bilgrami et al. 2020), and Denmark (*n* = 1) (Beuchert et al. 2016), while the other was a multi-country study taking place in eight European countries (Avendano et al. 2015). Eleven studies used a cross-sectional design (Chatterji and Markowitz 2005, 2008; Dundon et al. 2021; Feldman et al. 2004; Hwang et al. 2021; Kornfeind and Sipsma 2018; Mandal 2018; Shumbusho et al. 2020; Stack et al. 2018, 2019), eight were longitudinal studies (Clark et al. 1997; Dagher et al. 2014; des Rivières-Pigeon et al. 2001; Gjerdingen et al. 1991; Hyde et al. 1995; Jou et al. 2018; Klein et al. 1998; Petts 2018; Richman et al. 1991), and four followed a quasi-experimental design (difference-in-difference approach) (Avendano et al. 2015; Baker and Milligan 2008; Beuchert et al. 2016; Bilgrami et al. 2020). The tools used to assess depression varied across studies. The more commonly used were the CES-D (*N* = 9), the Edinburgh Postnatal Depression Scale (*N* = 4), and the PHQ-2 (*N* = 2). Details of each study, including statistical details, can be found in Supplementary file 2.

The mothers’ average age range within the included studies was 26 to 34 years. In twelve studies, more than half had education beyond high school (Beuchert et al. 2016; Bilgrami et al. 2020; Clark et al. 1997; des Rivières-Pigeon et al. 2001; Dundon et al. 2021; Feldman et al. 2004; Hwang et al. 2021; Hyde et al. 1995; Jou et al. 2018; Klein et al. 1998; Kornfeind and Sipsma 2018; Mandal 2018). Between 64 and 99% of mothers were married or had a partner in fifteen studies (Avendano et al. 2015; Beuchert et al. 2016; Bilgrami et al. 2020; Chatterji and Markowitz 2005; Dagher et al. 2014; des Rivières-Pigeon et al. 2001; Dundon et al. 2021; Hyde et al. 1995; Jou et al. 2018; Klein et al. 1998; Kornfeind and Sipsma 2018; Mandal 2018; Petts 2018; Stack et al. 2018, 2019), and this characteristic was an inclusion criterion in four (Feldman et al. 2004; Gjerdingen and Chaloner 1994; Clark et al. 1997; Hwang et al. 2021), while the other studies did not provide information about this. Between 10 and 51% were first-time mothers (Clark et al. 1997; Dagher et al. 2014; des Rivières-Pigeon et al. 2001; Dundon et al. 2021; Hyde et al. 1995; Klein et al. 1998; Kornfeind and Sipsma 2018), and this was an inclusion criterion in two studies (Feldman et al. 2004; Gjerdingen et al. 1991).

Nine studies provided details about the type of jobs the women had. Three studies explicitly focused on female medical residents (Stack et al. 2018, 2019; Dundon et al. 2021) and one on mothers in active military service (Shumbusho et al. 2020). The remaining five included women who worked as managers, professionals, technicians, services workers, sales workers, clerical workers, and in different blue-collar occupations (those that require hard manual labor) (Bilgrami et al. 2020; Chatterji and Markowitz 2005; Dagher et al. 2014; des Rivières-Pigeon et al. 2001; Gjerdingen et al. 1991).

The characteristics of the maternity leave policies in these studies are described in Table 1.

**Table 1:** Characteristics of maternity leave policies in the included studies

Location	Level of policy (national, state, private)	Maximum leave length (in weeks)	Payment scheme	Employment protection	Reference
Australia	National	18	Partially paid at minimum wage	Yes (2 years)	Bilgrami et al. 2020
Canada	National	Variable across states Pre-reform: 18–70 Post-reform: 50–70	Partially paid at 55%	Yes (1 year)	Baker and Milligan 2008 des Rivieres-Pigeon et al. 2001
Denmark	National	Pre-reform: 24^a^ (plus 52 additional weeks) Post-reform: 46^b^	Mixed scheme: Pre-reform: Fully paid: 24 weeks Partially paid at 60%: 52 weeks Post-reform: Fully paid	Not specified	Beuchert et al. 2016
European countries (Austria, Belgium, Denmark, France, Germany, Italy, Spain, Switzerland)	National	12–24 (variable across countries)	Paid in all countries, most to 100%	Yes (1 year)	Avendano et al. 2015
US	National	12	Unpaid^c^	Yes (12 weeks)	Chatterji and Markowitz 2005, 2008 Clark et al. 1997 Dundon et al. 2021 Feldman et al. 2004 Hyde et al. 1995 Jou et al. 2018 Klein et al. 1998 Kornfeind and Sipsma 2018 Mandal 2018 Petts 2018
US (specific to military workers)	National	Pre reform: 6 Post reform: 12	Paid^d^	Yes (not specified)	Shumbusho et al. 2020
Chicago area, US	City	12	Paid^d^	Yes (12 weeks)	Richman et al. 1991
Minnesota, US	State	6	Unpaid^c^	Yes (12 weeks)	Dagher et al. 2014 Gjerdingen et al. 1991
New York, US	State	12	Partially paid at 67%	Yes (12 weeks)	Hwang et al. 2021
US	Private	-	-	-	Stack et al. 2018, 2019

^a^14 weeks of maternity leave and 10 weeks of shared leave between both parents

^b^14 weeks of maternity leave and 32 weeks of shared leave between both parents

^c^Employers can choose to grant paid leave

^d^Conditions of paid leave are not specified

Seventeen studies explored if leave length affected maternal depression (Chatterji and Markowitz 2005, 2008; Clark et al. 1997; Dagher et al. 2014; des Rivières-Pigeon et al. 2001; Dundon et al. 2021; Feldman et al. 2004; Gjerdingen et al. 1991; Hyde et al. 1995; Klein et al. 1998; Kornfeind and Sipsma 2018; Mandal 2018; Petts 2018; Richman et al. 1991; Shumbusho et al. 2020; Stack et al. 2018, 2019). Six studies supported the negative association between leave length and depressive symptoms. Three US studies found that more extended maternity leave was significantly associated with lower scores or fewer cases of postpartum depression (Chatterji and Markowitz 2005, 2008; Dagher et al. 2014). Additionally, one US study identified that the likelihood of major depressive disorder was lower among mothers who returned to work after 12 weeks or more compared to mothers who returned within 12 weeks of giving birth (Mandal 2018), while another US study identified that taking between two or three months of leave lowers the probability of having depression compared to taking one month or less of leave (Petts 2018). Interestingly, one study in Montreal, Canada, found that women who were on maternity leave six months after childbirth had fewer depressive symptoms compared to homemakers and women seeking employment, but the association was not significant when compared to women who were back to work at that time (des Rivières-Pigeon et al. 2001). It was also observed that women with prenatal depression tended to take longer leaves, which was associated with a significant decrease in their depressive symptoms over time (Gjerdingen et al. 1991). One study found that although leave length was not significantly associated with postpartum depression, a combination of marital concerns (i.e. how concerned they are about their marital relationship) and leave length was associated with postpartum depression (Hyde et al. 1995). Although nine studies had non-significant results (Clark et al. 1997; Dundon et al. 2021; Feldman et al. 2004; Klein et al. 1998; Kornfeind and Sipsma 2018; Richman et al. 1991; Shumbusho et al. 2020; Stack et al. 2018, 2019), eight of them found a trend supporting an association between increased length and fewer symptoms or cases of postpartum depression.

Four studies explored the effects of wage replacement on depression (Bilgrami et al. 2020; Hwang et al. 2021; Jou et al. 2018; Mandal 2018). Two studies reported women having lower depressive symptoms when receiving paid leave than unpaid leave (Bilgrami et al. 2020; Mandal 2018), while the other two found no significant association.

Moreover, five studies investigated the effect of paid leave length on depression (Avendano et al. 2015; Baker and Milligan 2008; Beuchert et al. 2016; Chatterji and Markowitz 2008; Jou et al. 2018). One study showed that longer paid leaves are associated with fewer postpartum depressive symptoms and cases of severely depressed mothers (Chatterji and Markowitz 2008). Another study showed that longer full-wage leaves were associated with lower scores of depression in later life (Avendano et al. 2015). Although the other three studies did not find significant results, they align with the previous results showing that a more comprehensive leave is associated with reduced postpartum depression (Baker and Milligan 2008; Beuchert et al. 2016; Jou et al. 2018).

Finally, no studies explored the association between employment protection and postpartum depression.

The quality assessment of the articles showed that seven studies were categorized as strong, five as moderate and eleven as weak. Most weak studies had a cross-sectional design and inadequate control of confounders. Results from these studies were mixed. Details of the ratings can be found in Table 2.

**Table 2:** Quality assessment of the included articles

Study	Selection bias	Study design	Confounders	Blinding	Data collection methods	Withdrawals and drop-outs	Global rating
Avendano et al	2	2	1	1	1	2	**1**
Baker and Milligan 2008	2	2	3	1	3	3	**3**
Beuchert et al. 2016	1	2	1	1	2	2	**1**
Bilgrami et al. 2020	2	2	1	1	1	3	**2**
Chatterji and Markowitz 2005	2	2	1	1	1	2	**1**
Chatterji and Markowitz 2008	2	2	1	1	1	2	**1**
Clark et al	2	3	3	2	1	2	**3**
Dagher et al. 2014	2	2	1	2	1	2	**1**
des Rivieres-Pigeon et al. 2001	2	3	1	2	1	4	**2**
Dundon et al. 2021	3	3	3	1	1	4	**3**
Feldman et al. 2004	3	3	1	1	1	4	**3**
Gjerdingen et al. 1991	2	2	3	1	1	3	**3**
Hwang et al. 2021	2	3	1	2	1	4	**2**
Hyde et al. 1995	2	2	1	2	1	1	**1**
Jou et al. 2018	2	2	1	2	1	3	**2**
Klein et al. 1998	2	2	1	2	1	1	**1**
Kornfeid and Sipsma 2018	3	3	1	2	1	4	**3**
Mandal 2018	2	3	1	2	1	4	**2**
Petts 2018	2	3	1	2	3	4	**3**
Richman et al. 1991	2	3	3	2	1	4	**3**
Shumbusho et al. 2020	2	3	3	2	3	4	**3**
Stack et al. 2018	3	3	1	2	1	4	**3**
Stack et al. 2019	3	3	1	2	1	4	**3**

Legend: 1 = Strong quality; 2 = Moderate quality; 3 = Weak quality; 4 = Not applicable

## Discussion

To our knowledge, this is the first review focusing solely on the association between maternity leave and depression, which allows understanding in more depth how specific leave characteristics could have a differentiated effect on depression specifically. The review showed a trend suggesting that paid, longer maternity leaves are associated with fewer symptoms or cases of depression among mothers. However, these results are inconclusive, as only around half of the studies found significant results. Furthermore, there is a lack of studies focusing on how employment protection could be linked to postpartum depression. However, it is important to explore this further as it could influence maternity leave access and uptake.

Regarding leave length, six studies showed that more extended maternity leaves are linked with reduced postpartum depression, and two obtained similar results, although combining leave length with other variables, such as marital concerns and type of work. Also, while most studies showed associations between longer leaves and fewer postpartum depression symptoms, only one found the opposite. Notably, most studies finding a significant association were rated as having moderate to high quality. In contrast, most studies with no significant associations had low quality.

All studies exploring wage replacement in maternity leave were rated as having moderate quality and offered mixed results. When assessing wage and length combined, there is evidence that maternity leaves with more comprehensive conditions (i.e. longer, paid leaves) are associated with fewer symptoms of postpartum depression and potentially also in later life, which suggests a potential protective role in the long term. Thus, it seems that longer and paid maternity leave is a protective policy for the mother's mental health, especially considering other variables, such as socioeconomic status, that can influence leave uptake.

For example, in 2012, it was estimated that 23% of female workers in the US returned to work within ten days after labor due to financial reasons (Klerman et al. 2012), while one study in Taiwan showed that insufficient family income was associated with an increased risk of postpartum depression symptoms among immigrant and native women (Chien et al. 2012). Additionally, paid maternity leave is associated with maternal employment one year after labor (Vargas-Prada et al. 2018), which is positive since employment is a protective factor against depression (Heinz et al. 2018).

In general, and according to the ILO convention (International Labor Organization 2000), length and wage are variables that need to be considered together when analyzing and drafting maternity policies. Even though some mothers would prefer taking longer leaves, they might need to return to work earlier than desired, as they cannot afford to miss their salary. Therefore, it may explain why less privileged women are at higher risk of postpartum depression (Randhawa et al. 2021; Huynh et al. 2022) and use maternity leave less due to their financial limitations (Bingmer et al. 2020; Clark and Gallagher 2017; Guendelman et al. 2014; Rossin-Slater et al. 2013; Whitehouse et al. 2008).

Furthermore, it is crucial to consider that all the studies included in this review were conducted in high-income countries (HICs). Not only does this misrepresent the association between maternity leave and postpartum depression in low- and middle-income countries (LMICs), but it also misrepresents the social and demographic characteristics of mothers in the Global South. For example, the average age for the first pregnancy is between 20.9 and 22.8 years old in Africa, Latin America and the Caribbean, and Asia (Bongaarts et al. 2017), which is well below the average ages reported in the studies reviewed. In addition, educational levels beyond high school were common in the samples studied but are far less frequent in LMICs (Local Burden of Disease Educational Attainment Collaborators 2020), all of which could play a role in the mothers’ psychological health, access to maternity leave, and the association between maternity leave and postpartum depression, as well as having jobs with better working conditions.

Although there is still a sector of female workers with no access to maternity leave in HICs, such as US women working in small businesses with less than 50 employers, which hinders access to FMLA (Guendelman et al. 2014), the problem is significantly more common in LMICs, where informal work is widely extended (Huynh et al. 2022). According to the ILO, 2 billion people work in the informal sector worldwide, ranging from 25% in Europe and Central Asia to 60% in Latin America and the Caribbean (Basto-Aguirre et al. 2020) and 86% in Africa (International Labor Organization 2018), reaching 72% in Peru (Séruzier et al. 2014). In Latin America, women in the informal workforce are more prone to experiencing depression (Huynh et al. 2022). Unfortunately, this population is underrepresented in the reviewed studies. In order to grant informal working women and their children the economic and health benefits of maternity leave, governments could consider mechanisms to provide paid leaves to women in the informal workforce, an initiative already assessed theoretically in countries like Indonesia (Siregar et al. 2021).

Finally, we found that the included studies had strong, moderate and weak quality, reflecting the heterogeneity in methodologies across this field. Observational, cross-sectional studies were rated as having lower quality than quasi-experimental designs, and thus future research should follow stronger methodologies where possible. In addition to the study design, the included studies had issues related to selection bias and confounding variables. Longitudinal studies or difference-in-difference approaches with adequate rigor are feasible and capable of capturing the differences in maternal health associated with maternity leave policies.

### Limitations

One of the study limitations is language restriction. A possible result is that most studies referred to formally employed women in HICs, translating into less visibility of women from LMICs and those working informally. Moreover, most studies focused on postnatal leave and did not consider the implications of antenatal leave in their findings. As the desire to expand postnatal leave causes many women to work until the last days of pregnancy, exploring the antenatal period becomes necessary. Furthermore, while this study focused solely on maternity leave, paternity leave could be an important confounding variable that unfortunately has not been considered in the included studies. Thus, future studies could explore whether paternity leave also has an impact on the presence of postpartum depression. Finally, the heterogeneity and inadequate reporting of some results did not allow us to perform a meta-analysis, which would lead to more robust findings.

### Policy recommendations

Evidence suggests that longer paid leaves are associated with lower postpartum depression rates, while the opposite creates inequalities among mothers who can or cannot afford a secure income. Thus, universal coverage of maternity leave is essential to minimize inequalities (i.e., including mothers working in the informal sector and from vulnerable populations). Policy should be shaped in a manner that provides accessible and comprehensible maternity leave conditions to the general population, with adequate conditions for working mothers to access the maximum time allowed, with a secure income and employment protection.

## Conclusion

Evidence from HICs suggests that paid, longer maternity leave is associated with less postpartum depression. In this line, it would be recommended to continue exploring this topic in depth to ensure evidence is strong. In addition, the association between employment protection and postpartum depression needs to be explored, as well as the link between all maternity leave characteristics and postpartum depression in the Global South.

## Supplementary Information

Below is the link to the electronic supplementary material.

**Additional file 1: Supplementary material 1.** Search strategy.

**Additional file 2: Supplementary material 2.** Articles included in the review.
