# Supplementary material for: Association between maternity leave policies and postpartum depression: a systematic review
Source: Arch Womens Ment Health. 2023 Jul 17;26(5):571–80. doi: 10.1007/s00737-023-01350-z (PMC10491689; doi:10.1007/s00737-023-01350-z)
Supplement: Supplementary file 1 — Additional file 1: Supplementary material 1. Search strategy. [file 737_2023_1350_MOESM1_ESM.docx]

Association between maternity leave policies and postpartum depression: A systematic review

Archives of Women’s Mental Health

Liliana Hidalgo-Padilla*. CRONICAS Center of Excellence in Chronic Diseases, Universidad Peruana Cayetano Heredia, Lima, Peru**,**

Mauricio Toyama. CRONICAS Center of Excellence in Chronic Diseases, Universidad Peruana Cayetano Heredia, Lima, Peru**,**

Jessica Hanae Zafra-Tanaka. CRONICAS Center of Excellence in Chronic Diseases, Universidad Peruana Cayetano Heredia, Lima, Peru**,**

Alejandra Vives. Departamento de Salud Pública, Pontificia Universidad Católica de Chile, Santiago de Chile, Chile**,**

Francisco Diez-Canseco. CRONICAS Center of Excellence in Chronic Diseases, Universidad Peruana Cayetano Heredia, Lima, Peru

***** Corresponding author: [liliana.hidalgo.p@upch.pe](mailto:liliana.hidalgo.p@upch.pe)

**Supplementary material 1. Search strategy**

*Pubmed*

("Parental Leave"[Mesh] OR maternity leave OR paternity leave OR partner leave OR “ pregnancy leave”)

AND

("Depression, Postpartum"[Mesh] OR ((postpartum OR post-natal OR perinatal) AND (depres*)) OR ("Maternal Health"[Mesh] AND "Mental Health"[Mesh]) OR

((maternal OR postpartum) AND (mental health OR wellbeing OR distress)))

*Psycinfo*

((Index Terms: ("Employee Leave Benefits")) OR (Any Field: (maternity leave)) OR (Any Field: (parental leave)) OR (Any Field: (pregnancy leave))) AND ((IndexTermsFilt: ("Postpartum Depression")) OR (Any Field: (maternal) OR Any Field: (postpartum)) AND (Any Field: (mental health) OR Any Field: (wellbeing) OR Any Field: (distress)))

*EMBASE*

('maternity leave'/exp OR ((maternity OR paternity OR pregnancy) AND leave))

AND

('postnatal depression'/exp OR

((postpartum OR post-natal OR perinatal) AND (depression)) OR (('mental health'/exp OR 'psychological well-being'/exp OR 'emotional well-being'/exp) AND 'maternal welfare'/exp) OR ((mental health OR well-being OR distress) AND (maternal OR postpartum)))

*CENTRAL*

("Parental Leave"[Mesh] OR maternity leave OR paternity leave OR partner leave OR “ pregnancy leave”)

AND

("Depression, Postpartum"[Mesh] OR ((postpartum OR post-natal OR perinatal) AND (depres*)) OR ("Maternal Health"[Mesh] AND "Mental Health"[Mesh]) OR

((maternal OR postpartum) AND (mental health OR wellbeing OR distress)))

Filter: trials

*Global Index Medicus*

tw:(maternity leave OR paternity leave)
