# Supplementary material for: Association between maternity leave policies and postpartum depression: a systematic review
Source: Arch Womens Ment Health. 2023 Jul 17;26(5):571–80. doi: 10.1007/s00737-023-01350-z (PMC10491689; doi:10.1007/s00737-023-01350-z)
Supplement: Supplementary file 2 — Additional file 2: Supplementary material 2. Articles included in the review. [file 737_2023_1350_MOESM2_ESM.docx]

Association between maternity leave policies and postpartum depression: A systematic review

Archives of Women’s Mental Health

Liliana Hidalgo-Padilla*. CRONICAS Center of Excellence in Chronic Diseases, Universidad Peruana Cayetano Heredia, Lima, Peru**,**

Mauricio Toyama. CRONICAS Center of Excellence in Chronic Diseases, Universidad Peruana Cayetano Heredia, Lima, Peru**,**

Jessica Hanae Zafra-Tanaka. CRONICAS Center of Excellence in Chronic Diseases, Universidad Peruana Cayetano Heredia, Lima, Peru**,**

Alejandra Vives. Departamento de Salud Pública, Pontificia Universidad Católica de Chile, Santiago de Chile, Chile**,**

Francisco Diez-Canseco. CRONICAS Center of Excellence in Chronic Diseases, Universidad Peruana Cayetano Heredia, Lima, Peru

***** Corresponding author: [liliana.hidalgo.p@upch.pe](mailto:liliana.hidalgo.p@upch.pe)

**Supplementary material 2. Articles included in the review**

| **Author(s)** | **Publication year** | **Country** | **Study design** | **Population** | **Sample size** | **Comparison groups** | **Depression outcome measure** | **Results** | **Quality assessment rating** |
| --- | --- | --- | --- | --- | --- | --- | --- | --- | --- |
| Avendaño et al. | 2015 | Western Europe (Denmark, Austria, France, Germany, Belgium, Spain, Italy) | Quasiexperimental | European mothers born in 1960 or earlier who gave birth at 16 to 25 years of age | 495 | *Length of paid leave*   - Comprehensive leave: Women with 12 weeks of full-wage leave - Less comprehensive leave: Women with less than 12 weeks of full-wage leave | Euro-D | Reduction in depression score: -.385 (SD = .171) points **(p<.05)** Corresponds to a Cohen's d = .15 (small effect)  RR of depressive symptomatology comparing women with a comprehensive vs. a less comprehensive maternity leave = .82, 95% CI [.70, .96] | 1 |
| Baker & Milligan | 2008 | Canada | Quasiexperimental | Canadian children and mothers with a partner | 1674 | *Length of paid leave + lower eligibility criteria*   - Birth post-reform in Canada (25 weeks of paid leave) - Birth pre-reform in Canada (50 weeks of paid leave) | Depression index of 12 questions | Reduction in depression score (mean):  - 7-12 months = -.171 (SD = .238)  - 13-24 months = -.271 (SD = .254)  Regression coefficient: .272 (SD = .254) **(p>.05)** | 3 |
| Beuchert et al. | 2016 | Denmark | Quasiexperimental | Mothers who gave birth in the period 2 November 2001, to 1 March 2002 | 15494 | *Length of paid leave*   - Birth post-reform in Denmark - Birth pre-reform in Denmark | ICD-10 diagnosis and antidepressants purchase from clinical records | IV estimate (SD) = probability of (outcome) Within 1 year of childbirth Hospitalized with depression = -.002 (.010) **(p>.1)** Receiving antidepressants = -.001 (.017) **(p>.1)**  Within 3 years childbirth Hospitalized with depression = -.006 (0.014) **(p>.1)** Receiving antidepressants = .027 (0.027) **(p>.1)** | 1 |
| Bilgrami et al. | 2020 | Australia | Quasiexperimental | Mothers eligible for the Australian Paid Parental Leave (PPL) scheme | 1480 | *Wage replacement*   - Birth post-reform in Australia (paid leave) - Birth pre-reform in Australia (no paid leave) | Mental Health Index‐5 (MHI‐5) | IV Estimate of policy effect on depression severity = -.993 (SD = .392) **(p=.011)** … which translates to a 14% reduction in the likelihood of all depression (mild, moderate, and severe) | 2 |
| Chatterji & Markowitz | 2005 | USA | Cross-sectional | Women between 18 and 49 years old who had a pregnancy in 1988 and who had returned to work by the time the infant was six months | 1762 | *Leave length* (continuous) | CES-D (scores and cases) | IV Estimation of reduction in CES-D for each extra leave week (F test):  For depressive score: -.063 (-1.85) **p=0.000** For depressive case: -.018 (-1.22) **p=0.000** | 1 |
| Chatterji & Markowitz | 2008 | USA | Cross-sectional | Mothers who had worked during pregnancy and who had returned to work by the time of first follow-up interview, about 9 months after childbirth. | 3366 | *Leave length* (continuous)  *Length of paid leave* (continuous) | CES-D (scores and severe cases) | T statistic  Total number of weeks (paid or unpaid): Symptoms of depression: -.455 (-2.10) Severely depressed: -.062 (-1.54) **p=0.00**  Total number of paid weeks: Symptoms of depression: -.352 (-2.18) Severely depressed: -.050 (-1.62) **p=0.00** | 1 |
| Clark et al. | 1997 | Wisconsin, USA | Longitudinal | Pregnant women from Wisconsin | 570 | *Leave length* (continuous) | CES-D scores | Pearson correlation = -.05 **(not significant)** | 3 |
| Dagher et al. | 2014 | Minnesota, USA | Longitudinal | Adult women who delivered a healthy, singleton infant in three Minnesota hospitals in 2001 | 716 | *Leave length* (continuous) | Edinburgh Postnatal Depression Scale (EPDS) | 2SLS B = -.1075 (.0311) [-.1685;-.0465] **p≤.001** | 1 |
| Des Rivieres-Pigeon et al. | 2008 | Canada | Cross-sectional | Women who gave birth in four hospitals in Montreal in 1996 | 447 | *Leave length*  Employment status at 6 months postpartum 1. On maternity leave 2. Back to work 3. Homemakers 4. Unemployed/seeking employment | CES-D | ANOVA. Average CES-D score (SD) Women on maternity leave = 10.95 (8.49) Workers = 12.89 (10.01) Homemakers = 13.29 (9.90) Women seeking employment = 16.93 (11.13) **p=.009**  Mean difference (LSD) comparing women on maternity leave at 6m vs: Workers: -1.94 **(p=0.077)** Homemakers: -2.34 **(p=.046)** Women seeking employment: -5.98 **(p=.002)** | 2 |
| Dundon et al. | 2021 | USA | Cross-sectional | residents enrolled in pediatric residency programs | 18 | *Leave length*   - Short leave: ≤ 6 weeks - Longer leave: > 6 weeks | Edinburgh Postnatal Depression Scale (EPDS) (cases) | EPDS score > 9 Short leave: 50% Longer leave: 16.6% **p=0.15** | 3 |
| Feldman et al. | 2004 | USA | Cross-sectional | Mothers and fathers whose firstborn child was between 3 and 5 months old | 98 | *Leave length* (continuous) | Beck Depression Inventory | r -0∙24 **(p<0∙1)** | 3 |
| Gjerdingen et al. | 1991 | Minnesota, USA | Longitudinal | Married, first-time mothers. | 436 | *Leave length**   - less than 6 weeks - 6 weeks to 3 months - 3 to 6 months - more than 6 months   *on the basis of their response to the question included in each postpartum questionnaire: “Have you returned to work?” | The Hopkins Symptom Checklist | Maternity Leave Group depression mean scores (SD)  <6 weeks **(not significant)** Predelivery 11.7 (1.5) 6 Weeks Postpartum 12.5 (2.4) 3 Months Postpartum 13.5 (2.3) 6 Months Postpartum 11.2 (2.0)  6 weeks to 3 months **(not significant)** Predelivery 11.1 (2.6) 6 Weeks Postpartum 13.9 (3.1) 3 Months Postpartum 11.7 (2.9) 6 Months Postpartum 13.3 (4.8)  3 months to 6 months **(not significant)** Predelivery 12.8 (2.5) 6 Weeks Postpartum 13.8 (3.9) 3 Months Postpartum 12.2 (2.8) 6 Months Postpartum 11.7 (1.9)  >6 months (t test **p<.01**) Predelivery 14.5 (2.4) 6 Weeks Postpartum 13.9 (3.6) 3 Months Postpartum 12.7 (3.3) 6 Months Postpartum 12.1 (2.5) | 3 |
| Hwang et al. | 2020 | New York, USA | Cross-sectional | Employed mothers in dual income couples who were eligible to take paid leave and who returned to work after childbirth. | 92 | *Wage replacement*   - Mothers who used unpaid leave only - Mothers who used some or all paid leave | CES-D | Hierarchical Regression Model Paid leave use: B=.04 (.09) **(not significant)** | 2 |
| Hyde et al. | 1995 | Wisconsin, USA | Longitudinal | Adult pregnant women living with a partner from two clinics and one hospital specializing in care for low-income women in Wisconsin | 570 | *Leave length* (continuous) | CES-D | Longitudinal regression analysis Model 4 B = -0.066 **(not significant)**  *Significant when combined with marital concerns (B=-.474, **p<.05)** | 1 |
| Jou et al. | 2018 | USA | Longitudinal | Women aged 18–45 who gave birth to singleton infants in U.S. hospitals from July 2011 to June 2012 | 700 | *Wage replacement*   - Women who took partially- or fully-paid leave - Women who did not use paid leave - Women who took only unpaid leave   *Length of paid leave*   - 1-6 weeks - 7-12 weeks - >12 weeks | Patient Health Questionnaire-2 (PHQ-2) | Wage replacement effect on depressive symptoms (Adjusted Odds Ratio) **(not significant)** Used paid leave (AOR=1.0) compared to Did not use paid leave (AOR=.88) [.55 – 1.42] Used paid leave (AOR=1.0) compared to Used unpaid leave only (AOR =.87) [.48 – 1.57]  Length of paid leave effect on depressive symptoms (Base=0weeks) (Adjusted Odds Ratio) **(not significant)** 1–6 weeks: AOR =1.15 [.63 – 2.11] 7–12 weeks: AOR =1.17 [.61 – 2.25] 12+ weeks: AOR =.25 [.05 – 1.23] | 2 |
| Klein et al. | 1998 | USA | Longitudinal | Women one year after delivery | 437 | *Leave length* (continuous) | CES-D | Regression Analysis: Depression at 4 months postpartum and length of leave: B=.002 **(not significant)** | 1 |
| Kornfeind & Sipsma | 2018 | USA | Cross-sectional | Women who gave birth to a single baby from July 2011 to June 2012 in a US hospital | 177 | *Leave length*   - Mothers with ≤ 12 weeks of leave - Mothers with > 12 weeks leave | PHQ-2 | Odds ratio of depressive symptoms in the last 2 weeks Total population: OR = .90 [.80-1.01] **(not significant)** Shorter leave: OR = .58 [.40-.84] **(p<.01)** Longer leave: OR = .97 [.73-1.29] **(not significant)** | 3 |
| Mandal | 2018 | USA | Cross-sectional | Women who worked full-time pre-birth, and were not self-employed, and had singleton births | 3850 | *Leave length*   - Return to work at 9 months - Return to work in 12 weeks vs after 12 weeks   *Wage replacement*   - Some paid leave vs no paid leave (returned to work at 9 months, in 12 weeks; returned to full-time work at 9 months, in 12 weeks) | CES-D | PSM for CESD scores; OR for Binary CESD  Leave length Return to work at 9 months: PSM = -.148 (.055) **p<.01** OR = .901 (.083) **not significant** Return to work in 12 weeks vs after 12 weeks: PSM = .131 (.053) **p<.05** OR = 1.217 (.117) **p<.05**  Wage replacement Some paid leave vs no paid leave of those who returned to work at 9 months PSM = -.123 (.061) **p<.05** OR = .760 (.073) **p<.01** Some paid leave vs no paid leave of those who returned to work in 12 weeks PSM = -.167 (.074) **p<.05** OR = .637 (.079) **p<.01** Some paid leave vs no paid leave of those who returned to full-time work at 9 months PSM = -.181 (.079) **p<.05** OR = .757 (.089) **p<.05** Some paid leave vs no paid leave of those who returned to full-time work at 9 months in 12 weeks PSM = -.321 (.100) **p<.01** OR = .668 (.096) **p<.01** | 2 |
| Petts | 2017 | USA | Longitudinal | Fragile families: unmarried parents and their children interviewed in both the baseline (interviewed shortly after the child’s birth) and 1-year follow-up surveys | 1726 | *Leave length*   - ≤ 1 month - 2-3 months - 4-6 months - > 6 months - did not return to work | CIDI-Short Form | Regression Models for depression 1 month or less vs: 2-3 months: OR = .64 (.12) **p<.05** 4-6 months: OR = .82 (.18) **not significant** > 6 months: OR = .63 (.17) **p<.10** did not return to work: OR = .71 (.20) **not significant** | 3 |
| Richman et al. | 1991 | USA | Longitudinal | Married couples who were at least 18 years old, expecting their first child and had no major health problems | 84 | *Leave length*   - Full-time work at 8 weeks - On Maternity leave at 8 weeks | CES-D | Depressive mood mean scores (SD) **not significant** Full-time work at 8 weeks: 12.60 (8.54) On Maternity leave at 8 weeks: 9.42 (7.63) | 3 |
| Shumbusho et al. | 2020 | USA | Cross-sectional | Active duty service members who gave birth | 214 | *Leave length*   - 6 weeks - 12 weeks | Self-reported question | Fisher exact test % of participants with postpartum depression 6 weeks: 16.1% 12 weeks: 9.5% **p=.11** | 3 |
| Stack et al. | 2018 | USA | Cross-sectional | Female medical residents from the University of Washington who took maternity leave during their residency and were 12 months or less postpartum | 13 | *Leave length*   - <8 weeks - ≥8 weeks | Edinburgh Postnatal Depression Scale (EPDS) | % of participants with NO postpartum depression <8 weeks: 33% ≥8 weeks: 70% **p=0.51** | 3 |
| Stack et al. | 2019 | USA | Cross-sectional | Female medical residents enrolled across 6 universities who took maternity leave during their residency | 77 | *Leave length*   - <6 weeks - ≥6 weeks | Edinburgh Postnatal Depression Scale (EPDS) | % of participants with NO postpartum depression <6 weeks: 87.5% ≥6 weeks: 84.2% Delta: -.03 [-.63-.57) **p=0.76** | 3 |
